# Changes in Soft-Tissue Sarcoma Treatment Patterns over Time: A Population-Based Study in a Country with Universal and Centralized Healthcare

**DOI:** 10.1155/2019/8409406

**Published:** 2019-09-16

**Authors:** Anthony Bozzo, Hsien Seow, Gregory Pond, Michelle Ghert

**Affiliations:** ^1^Division of Orthopaedic Surgery, Department of Surgery, McMaster University, Hamilton, Ontario, Canada; ^2^Institute for Clinical and Evaluative Science (ICES), McMaster University, Hamilton, Ontario, Canada; ^3^Department of Oncology, McMaster University, Hamilton, Ontario, Canada; ^4^Hamilton Health Sciences, Juravinski Hospital and Cancer Center, Hamilton, Ontario, Canada

## Abstract

**Background:**

The clinical care of soft-tissue sarcoma (STS) patients is largely multidisciplinary involving clinicians from surgical disciplines, medical oncology, and radiation oncology. It is not clear if treatment patterns for STS have changed over time. We present population-level data on changes in treatment patterns of patients diagnosed with STS of all stages in Ontario, Canada.

**Methods:**

We performed a population-based cohort study using linked administrative databases in Ontario, Canada, of patients with STS between 2006 and 2015. Patients with the AJCC stage at the time of diagnosis were included. Patients were categorized into one of the seven treatment arms: single modality treatment (surgery, chemotherapy, or radiation therapy), bimodality therapy, or all three treatment modalities. Survival of STS patients of different stages is displayed with the Kaplan–Meier method.

**Results:**

A total of 4696 patients were diagnosed with biopsy-proven sarcoma during the study period including 1915 patients with stage information available. Treatment patterns for patients with Stage 1 and 2 disease were similar enough to allow for grouping. The use of radiation therapy in Stage 1 and 2 patients increased by 15% over the study period. None of the 7 treatment regimens for Stage 3 patients changed appreciably during the study period. We observed that the use of chemotherapy for Stage 4 STS patients increased 36% during the study period. Overall patient survival was, as expected, highest in Stage 1 patients and lowest in Stage 4 patients.

**Conclusion:**

This is the first population-level study reporting of 7 different STS treatment regimens in a country with universal and centralized healthcare. Radiation therapy for local disease control and chemotherapy for Stage 4 patients have recently become more utilized. Survival from STS is highly dependent on stage at presentation. Other population-based studies from other countries are needed to establish the current international treatment patterns.

## 1. Background

Sarcomas are rare malignancies constituting less than 1% of all adult cancers, and there are over 50 soft-tissue sarcoma (STS) and bone sarcoma subtypes [1]. Management of sarcoma is multidisciplinary and may involve surgery with wide resection, neoadjuvant or adjuvant chemotherapy, and preoperative or postoperative radiation, in varied combinations [2, 3].

Recently, large population-based observational studies of STS patients have become popular as they can capture more patients than controlled study designs and can provide valuable information regarding long-term outcomes [4–6]. Thus far, studies derived from population-based administrative databases, such as the Surveillance, Epidemiology, and End Results (SEER) database in the USA, have provided incidence rates for specific sarcoma subtypes [7, 8] along with outcome data for up to 10 years [9]. These studies have characterized differences between pediatric and adult patients in sarcoma subtype prevalence and location of the disease [10] and characterized outcome differences based on race and gender [11]. However, the treatment regimens of sarcoma patients have not been assessed at the population level [8, 12, 13].

Generally, STS is a disease treated with surgery and radiation therapy [14]. Routine use of chemotherapy is not supported as several key trials failed to show survival benefits [15, 16]; however, the 2018 European Society of Medical Oncology (ESMO) guidelines allow for the use of chemotherapy in STS patients, often in cases of advanced disease or for palliation [3]. Treatment regimens are usually based on the clinical stage (tumour grade, tumour size, and presence of lymph node or distant metastases) and can be broadly classified into seven categories: surgery alone, radiation therapy alone, chemotherapy alone, three bimodal combinations, and lastly the combination of all three modalities. The use of multimodal therapy is generally associated with higher stages of disease. At a patient-specific level, comorbidities, age, and patient preferences also contribute to treatment decisions. To our knowledge, no other population-based studies to date have assessed the overall treatment patterns of sarcoma patients in a country with universal and centralized healthcare for treating sarcoma patients.

The purpose of this study was to investigate a large population-based database of sarcoma patients collected over the past 10 years in order to determine the treatment regimens provided for STS patients of different stages and if treatment regimens have changed over time. We also investigated overall survival based on the stage of the disease.

## 2. Methods

### 2.1. Study Design and Population

We performed a population-based cohort study using linked administrative databases in Ontario, Canada, in accordance with RECORD guidelines which extend the STROBE guidelines for observational studies to administrative healthcare data [17, 18]. All patients with biopsy-confirmed diagnosis of sarcoma between January 1, 2006, and December 31, 2015, were eligible. The International Classification of Diseases, 10th Edition (ICD-10), Clinical Modification diagnosis codes for all STS subtypes were used for classification. As per prior research, we excluded diseases with a considerably different diagnosis, management, and prognosis such as Kaposi, visceral, bone, and uterine sarcomas, gastrointestinal stromal tumors, and mesotheliomas [19]. Only patients with American Joint Committee on Cancer (AJCC) stage information were used to determine stage-specific treatment patterns. See Supplementary Materials ([Supplementary-material supplementary-material-1]) for details of the codes and to identify patients and their treatments.

### 2.2. Data Sources

Data were obtained from the Institute for Clinical Evaluative Sciences (ICES). ICES holds several provincial health care administrative databases and links them together via encrypted health insurance number of Ontario residents. The person-level linking of all these databases allows for a comprehensive longitudinal follow-up of a patient's interactions with the healthcare system. Databases used include the Ontario Cancer Registry which provides the biopsy-confirmed diagnostic information, the Discharge Abstract Database which contains information on hospitalizations, surgical procedures, and other treatment data, and the Cancer Activity Level Reporting database which contributes information regarding chemotherapy and radiation therapy. Databases containing information on physician billings (Ontario Health Insurance Plan), emergency department visits (National Ambulatory Care Reporting System), prescription medications (Ontario Drug Benefit), and death (Registered Persons Database) were also linked. Using these databases, we collected demographic information including sex, age at surgery, subtype of sarcoma, place of residence, income quintile, chemotherapy and radiation therapy treatment information, vital status at time of data collection, and Charlson–Deyo Comorbidity Index [20, 21]. Physician billing codes in these databases have been validated in the measure of other conditions such as heart disease and diabetes [22–24].

### 2.3. Statistical Methods

Demographic data and treatment patterns are summarized using descriptive statistics. Patients were categorized by treatment received as having single modality treatment (surgery, radiation therapy, or chemotherapy), bimodality therapy, or all three treatment modalities. Treatments are included if they occurred within 1 year of diagnosis. As the treatment patterns for patients with Stage 1 and 2 disease were quite similar, we grouped these stages together for presentation. We present changes in the treatment patterns of patients from the first five-year period of our cohort (2006–2010) to the second five-year period (2011–2015).

All statistical analyses were performed with R version 3.3.0 (http://www.r-project.org) [25] and Microsoft Excel 2016. The authors AB and GP had direct access to the data. Cell sizes of 5 or less are reported as “<6” as per ICES guidelines. Ethical approval was provided for this study by the Hamilton Integrated Research Ethics Board (HiREB) for observational research with encrypted and anonymized patient information (REB# 3745-C).

## 3. Results

We identified 4696 patients with biopsy-confirmed STS diagnosis during the study period. A total of 1915 STS patients (40.8%) had AJCC stage information available. Patient characteristics of the entire cohort are summarized in Table 1. There is a near 1.5 : 1 ratio of males to females in our cohort, and 68% of STSs occurred in patients of 50 years of age or above. Sarcoma cases were evenly distributed among income quintiles, and 13.1% of patients were living in rural areas. There was a 23% increase in the number of STS cases with stage information between the first and the second half of the study period. A total of 57 STS subtypes were identified within our database, and the full list is available in Supplementary Materials ([Supplementary-material supplementary-material-1]).

### 3.1. Sarcoma Treatment for Stage 1 and 2 Patients

**Figure 1:**
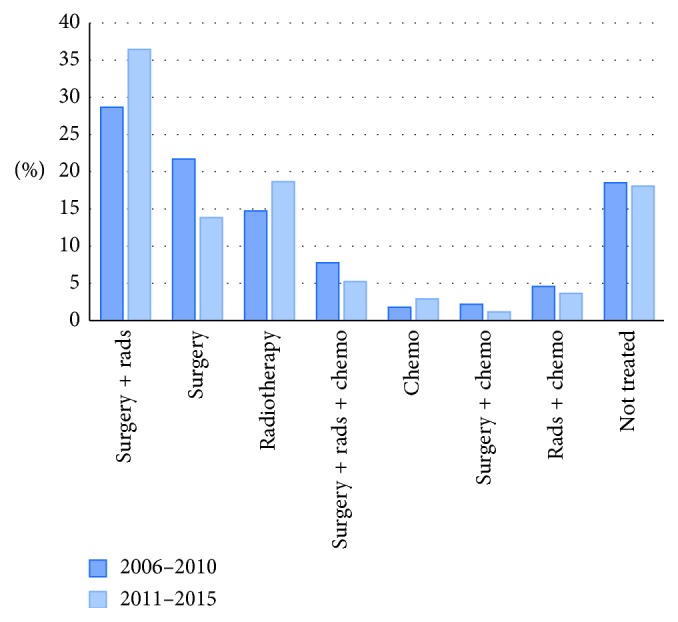
Treatment of Stage 1 and 2 STS patients. There are *N* = 1188 patients with complete stage information. Rads: radiotherapy; chemo: chemotherapy.

Treatment patterns for patients with Stage 1 and 2 disease (localized low-grade to midgrade tumours) were alike enough to allow for grouping. The combination of surgery and radiation therapy was the most common treatment regimen for Stage 1 and 2 patients, and complete treatment information is presented in Figure 1. Of note, we observed a 15% relative increase in the use of radiation therapy in the most recent 5 years compared to the first half of our study period. While 55% of Stage 1 and 2 patients received radiation therapy from 2006 to 2010, 64% received radiation therapy from 2011 to 2015. Preoperative radiation therapy for STS was initiated at a median of 33 days from biopsy. In patients receiving radiation therapy, surgery occurred at a median of 115 days from biopsy.

### 3.2. Sarcoma Treatment for Stage 3 Patients

**Figure 2:**
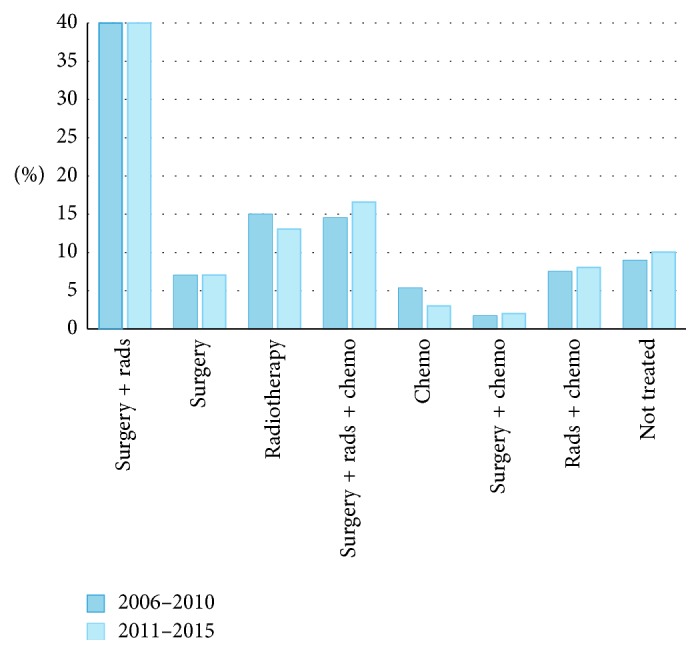
Treatment of Stage 3 STS patients. There are *N* = 414 patients with complete stage information. Rads: radiotherapy; chemo: chemotherapy.

Detailed treatment information for STS patients with Stage 3 disease, who generally present with high-grade, large tumors without distant metastases, is presented in Figure 2. Just over 40% of Stage 3 STS patients were treated with the combination of surgery and radiation therapy, and all treatment patterns remained remarkably similar between each half of our study period. This group had the lowest proportion of patients receiving no treatment, 8.9%. A total of 29% of Stage 3 patients received chemotherapy in any combination of treatments.

### 3.3. Sarcoma Treatment for Stage 4 Patients

**Figure 3:**
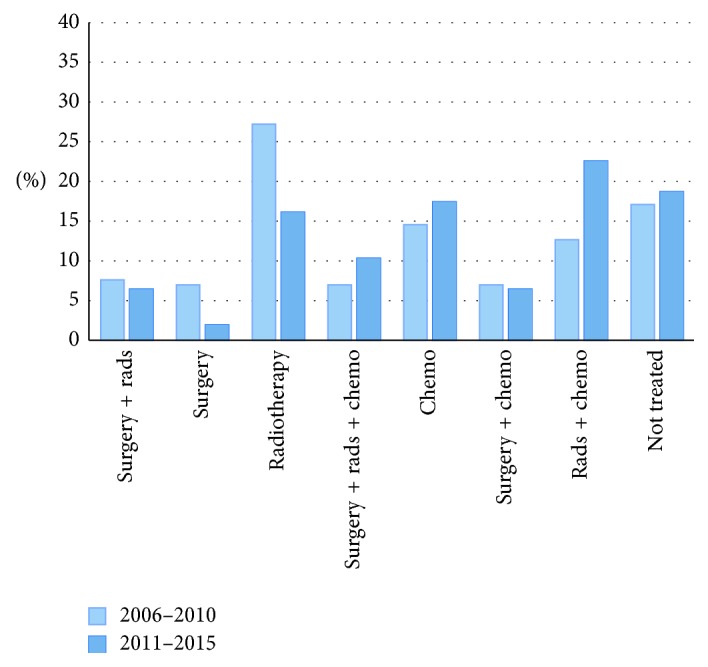
Treatment of Stage 4 STS patients. There are *N* = 313 patients with complete stage information. Rads: radiotherapy; chemo: chemotherapy.

Detailed treatment information for STS patients with Stage 4 (metastatic) disease is presented in Figure 3. In contrast with the other groups, 49% of patients with Stage 4 STS received chemotherapy. Considering only the most recent 5 years, 57% of STS patients received chemotherapy, a relative increase of 36% from the use of chemotherapy in the first 5 years of the study period. Only a minority of Stage 4 patients were treated with surgery and radiation (7%), the most common treatment regimen for all other stages. About 18% of Stage 4 patients did not receive surgical or systemic treatment.  Table 2 provides the treatment patterns over the entire study period for patients with and without stage information.

### 3.4. Survival by Stage

**Figure 4:**
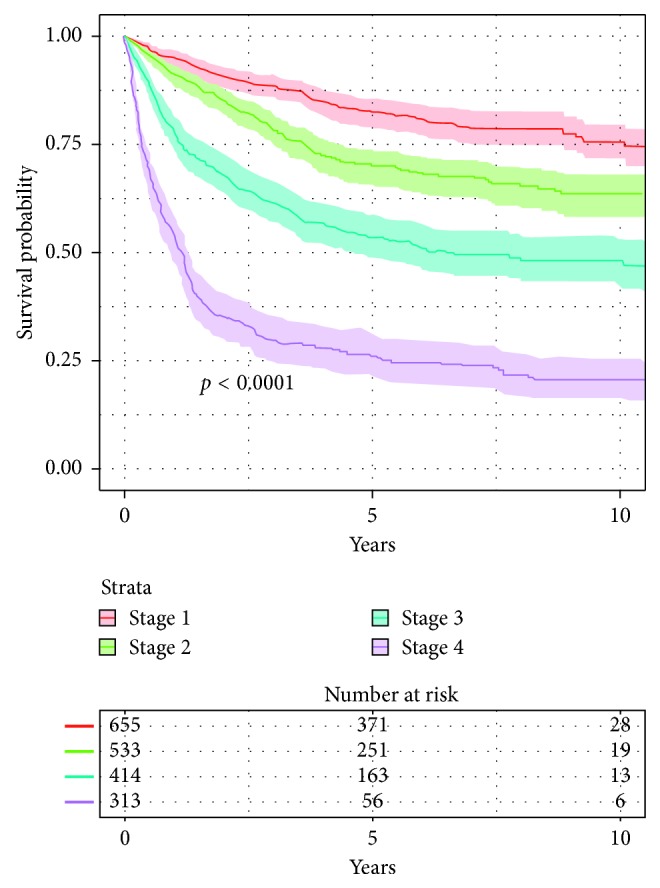
Overall survival after STS diagnosis, by stage. Significantly different survival is seen for STS patients presenting at different stages (log-rank test: *p* < 0.0001).

Overall survival following diagnosis of Stage 1 STS was 82% at 5 years and 74% at 10 years. Overall survival following bone sarcoma diagnosis was 68% at 5 years and 61% at 10 years. Stage 3 patients displayed 51% survival at 5 years and 45% at 10 years, while Stage 4 patients showed only 19% survival at 5 years and 13% at 10 years. Accordingly, the median survival for Stage 4 patients is 0.96 years (IQR: 0.74–1.16), while it is considerably longer at 5.4 years (IQR: 3.7–NA) for Stage 3 patients. As more than 50% of Stage 1 and 2 patients lived to the end of the follow-up period, median survival is not calculable in those groups. The Kaplan–Meier survival curves for STS patients by stage at initial presentation are presented in Figure 4.

## 4. Discussion

Our paper is the first to provide data on population-level treatment regimens for local and metastatic STS in a country with universal and centralized healthcare for sarcoma treatment and the first to demonstrate how treatment patterns may change. The combination of surgery and radiation therapy is the mainstay of treatment for STS patients with Stage 1, 2, or 3 disease at presentation, and the use of radiation therapy in patients with Stage 1 and 2 disease increased by 15% in the last 5 years. Furthermore, the use of chemotherapy in Stage 4 patients increased by 36% over the course of our study period while remaining unchanged in patients of other stages. Our reported prevalence of the most common sarcoma subtypes and the observed 1.5 : 1 male-to-female predilection are similar to those in other population-based studies [10, 26, 27].

The use of chemotherapy for STS patients is controversial but has been studied for many decades. Initial trials in the 1970s and 1980s failed to demonstrate survival benefits from the use of doxorubicin alone, while later trials demonstrated some advantages from the combination of doxorubicin and ifosfamide [28]. A systematic review which included 4 newer trials from 2000 to 2002 as well as 14 RCTs from 1977 to 1987 found a small but significant reduction in the mortality risk of 6% (95% CI: 2–11%) from the use of any chemotherapeutic regimen [29]. Several recent large international multicenter RCTs have been conducted with more emphasis on the selection of drugs, patients, doses, and sequence: the 2012 EORTC trial compared the use of doxorubicin and ifosfamide to no chemotherapy and failed to show a difference in survival [15], and the 2014 EORTC study published in The Lancet showed that ifosfamide and doxorubicin did not provide significant survival benefit compared to doxorubicin alone [30]. A pooled analysis of two EORTC trials failed to demonstrate a survival benefit in young patients or other subgroups [16]. While the 2016 trial published in The Lancet did show that the combination of olaratumab with doxorubicin conferred STS patients with locally advanced or metastatic disease an additional 11.8 months of overall survival compared to doxorubicin alone [31], the 2017 trial published in the same journal showed no benefit to tailoring the chemotherapeutic regimen to the histologic subtype [32]. Despite the lack of convincing evidence of the effect of chemotherapy on overall survival, we observed the use of chemotherapy in 29% of Stage 3 patients and 49% of Stage 4 patients. The use of chemotherapy is likely for adjuvant or palliative purposes [33].

Recently, data-driven apps such as SARCULATOR from Milan and PERSARC from Leiden have provided prognostication for local recurrence and overall survival following STS resection and treatment [34, 35]. Both apps generally display increased survival and lower chance of local recurrence for stage II sarcomas treated with radiation therapy—benefits which are concordant with our observed ones increased usage of this treatment modality. To our knowledge, the only other study reporting population-level treatment information is a Scandinavian study published in 2001. While the authors do not report detailed treatment regimens, they state that only 4% of their STS patients received chemotherapy [36] during a time when there were no national guidelines on the use of chemotherapy for STS patients. Of note, studies have demonstrated better adherence to sarcoma treatment guidelines for patients referred to specialized tertiary sarcoma centres [37, 38], and care for sarcoma patients in our country is highly centralized. Additional updated population-based studies from several countries are needed to replicate our findings of the popular use of chemotherapy in Stage 4 STS patients and to determine what treatment regimens constitute the current international standard in the management of advanced STS. While it is challenging with healthcare administrative data to identify the specific chemotherapeutic agents utilized, and whether the goal of treatment was curative or palliative, further exploration of these topics may represent fruitful areas for future research.

Our study has several strengths: Firstly, administrative records of healthcare use are unaffected by recall bias and provide large, general population samples and information on long-term follow-up. By virtue of including all sarcoma patients with stage information over a 10-year period, our analyzed sample closely mirrors the intended population. We can therefore place more confidence in the generalizability of our results to future Ontario sarcoma patients. While STS is a heterogeneous group of tumors, we excluded sarcomas most likely to not be representative of general treatment and prognostic characteristics.

### 4.1. Limitations

This is an observational study that does not demonstrate causation. Although AJCC stage information is available for over 40% of patients as of 2006, it was recorded in less than 2% of patients in the preceding years, limiting the long-term understanding of the effect of stage on outcomes. While tumour grade is not a variable collected in our database, grade is incorporated and reflected in AJCC staging. Reporting is likely to continue to improve with time [39], and future analyses will be able to incorporate a greater number of well-reported important variables. While the AJCC staging system has changed subtly [40], our data capture the stage according to the criteria at the time of biopsy. Likewise, the condition formerly known as malignant fibrous histiocytoma is now named “undifferentiated pleomorphic sarcoma,” but both use the same ICD-10 code, and we report the disease as originally labelled in the database.

Specific threats to validity for studies using administrative data are described in the literature; misclassification of data is known to occur [41], and the concept of accuracy encompasses 5 additional subcomponents including *completeness*, *correctness*, *measurement error*, *internal consistency*, and *temporal consistency* [42]. However, the data provided by ICES include information on how many variables are missing, if any, for each field. Several validation studies have been performed on ICES data by comparing the ICES diagnoses with data collected directly from patient charts and determined a specificity of at least 94% for ICES diagnoses of arrhythmia, congestive heart failure, or unstable angina [22, 43]. While no validation studies have been performed for sarcoma patients, we expect a high relative accuracy given that the diagnostic codes used to identify sarcoma patients are based strictly on biopsy and a diagnosis from a pathologist—stringent criteria with little to no room for interpretation. Thus, we expect the patients identified with ICD-10 codes to truly have a diagnosis of sarcoma.

## 5. Conclusion

This population-based cohort study presents the multidisciplinary treatment regimens and demographic information of soft-tissue sarcoma patients treated in a single-payer universal healthcare system for over 10 years. The use of radiation therapy in Stage 1 and 2 patients has increased 15% and the use of chemotherapy in Stage 4 patients has increased 36% over the study period. Other population-based studies are needed to provide an international overview of treatment patterns for sarcoma patients.

## Figures and Tables

**Table 1:** Demographic information of soft-tissue sarcoma patients.

Characteristics	2006–2010		2011–2015	
Total Ontario sarcoma patients	2217		2479	
Age group				
<35	310	14.0%	269	10.9%
35–49	396	17.9%	392	15.8%
50–59	362	16.3%	436	17.6%
60–69	396	17.9%	492	19.8%
70–79	386	17.4%	470	19.0%
80+	367	16.6%	420	16.9%

Gender				
Female	942	42.5%	1050	42.4%
Male	1275	57.5%	1429	57.6%

Most common subtypes				
Liposarcoma^¥^	356	16.1%	518	20.9%
Malignant fibrous histiocytoma	250	11.3%	145	5.8%
Leiomyosarcoma	240	10.8%	300	12.1%
Giant-cell sarcoma	91	4.1%	189	7.6%
Fibromyxosarcoma	66	3.0%	165	6.7%

Topography (ICD topography code)				
Lower limb (C40.2, C49.2)	678	30.6%	809	32.6%
Upper limb (C40.0, C40.1, C49.1)	294	13.3%	311	12.5%
Axial	1245	56.2%	1359	54.8%

Charlson–Deyo comorbidity score (1–18)				
Median	3.0		3.0	
Mean	3.7		3.6	

Stage				
I	264	11.9%	391	15.8%
II	238	10.7%	295	11.9%
III	199	9.0%	215	8.7%
IV	158	7.1%	155	6.3%
Not reported	1356	61.3%	1423	57.4%

Income quintile^ˠ *σ*^				
Lowest	401	18.1%	396	16.0%
2^nd^	415	18.7%	463	18.7%
3^rd^	417	18.8%	499	20.1%
4^th^	470	21.2%	561	22.6%
Highest	505	22.8%	546	22.0%

Place of residence^*σ*^				
Urban	1917	86.5%	2195	88.5%
Rural	298	13.4%	281	11.3%

See Appendix for the full list of sarcoma subtypes. ^ˠ^Based on nearest neighborhood census information. ^*σ*^Proportion of missing data is 0.1% for place of residence and 0.3% for income quintile. ^¥^Liposarcoma subtypes include “dedifferentiated,” “pleomorphic,” “round cell,” “mixed,” and “NOS.”

**Table 2:** Sarcoma treatment regimens by stage of disease, 2006–2015.

	Stages 1 and 2	Stage 3	Stage 4	Unknown stage
Total patients	1188	414	313	2779
Surgery + radiation therapy (%)	33.2	40.1	7.0	22.6
Surgery (%)	17.2	7.0	4.5	13.1
Radiation therapy (%)	17.0	15.0	21.7	9.6
Surgery + radiation therapy + chemotherapy (%)	6.3	14.5	8.6	3.7
Chemotherapy (%)	2.4	5.3	16.0	6.7
Surgery + chemotherapy (%)	1.6	1.7	6.7	3.6
Chemotherapy + radiation therapy (%)	4.0	7.5	17.6	4.3
No reported treatment (%)	18.3	8.9	17.9	36.3

## Data Availability

ICES data are provincial data that are protected by the government. Access is normally given only to ICES employees and authorized researchers.

## Supplementary Materials

Supplementary Materials Table S1: summary of codes used.
