# Supplementary material for: Changes in Soft-Tissue Sarcoma Treatment Patterns over Time: A Population-Based Study in a Country with Universal and Centralized Healthcare
Source: Sarcoma. 2019 Sep 16;2019:8409406. doi: 10.1155/2019/8409406 (PMC6766085; doi:10.1155/2019/8409406)
Supplement: Supplementary Materials — Table S1: summary of codes used. [file 8409406.f1.pdf]

Supplementary Material

Summary of codes used

Variable	ICD10 Code	N unique
Chemotherapy<br><br>-Presence of these codes in the OHIP, as well as matching patient IDs in the ALR-Chemo database	G281<br>G339<br>G359<br>G381<br>G382<br>G388<br>Z511<br>Z512	3905
Surgery<br><br>- Presence of these codes in the OHIP database, within 1 year of biopsy confirmed sarcoma diagnosis	Surgical codes: R037, R214, R216, R226, R246, R253, R266, R272, R293, R294, R295, R297, R330, R523, R591, R592, R641<br><br>Amputation codes: R614, R616, R620, R630, R631<br>Tumor excision codes: Z632, Z633, Z634<br>Retroperitoneal tumor: S431<br>Radical Soft Tissue Tumour Excision: N554, N553	5294
Radiotherapy<br><br>-Presence of these codes in the OHIP database, as well as matching patient IDs in the ALR-Rads database	X310<br>X311<br>X312<br>X313	4289
